# Apolipoprotein L1 genetic testing, family history of hypertension, and kidney disease in a Midwestern U.S. cohort

**DOI:** 10.3389/fneph.2026.1771125

**Published:** 2026-05-25

**Authors:** Krista L. Lentine, Rengin Elsurer Afsar, Bryan Clair, Aliza Anwar Memon, John C. Edwards, Kana N. Miyata, Baris Afsar, Mark Schnitzler, Huiling Xiao, Amber Carriker, Fadee Abu Al Rub, Chi-yuan Hsu, Anthony N. Muiru, Barry I. Freedman, Marie D. Philipneri, Yasar Caliskan, Orhun Aydin, Ezequiel Bellorin, Ava DeLonais-Parker, Nourhan Houjeij, Kevin Lee, Kunal Malhotra, Rushda Mansuri, Marilyn Maxwell, Mowafaq Said, Farzana Hoque, Amy K. Mosman, Anne Buganski, Mary Lesko, Kenan Li, Enbal Shacham, Than-Mai Vo

**Affiliations:** 1 Saint Louis University School of Medicine, St. Louis, MO, United States; 2 SSM Health Saint Louis University Hospital, St. Louis, MO, United States; 3 Department of Mathematics, Saint Louis University, St. Louis, MO, United States; 4 Mid-America Transplant, St. Louis, MO, United States; 5 University of California, San Francisco, San Francisco, CA, United States; 6 Wake Forest University School of Medicine, Winston-Salem, NC, United States

**Keywords:** African American, apolipoprotein L1, cohort, family history, genetics, hypertension, kidney disease

## Abstract

**Background:**

Understanding how family history and genetic factors—particularly apolipoprotein L1 (*APOL1*) renal risk variants (RRVs)—contribute to kidney disease risk among Black individuals continues to evolve.

**Methods:**

In a cohort of prospectively enrolled Black adults who underwent *APOL1* genotyping and completed surveys at a Midwestern U.S. academic hospital (01/24/2019–03/21/2025; NCT05656261), we examined *APOL1* RRV distribution in relation to family history, baseline clinical factors, and 3-month renal function change.

**Results:**

Among 220 eligible participants, 17% (37/220) carried *APOL1* high-risk genotypes (two RRVs). Family history of hypertension alone was reported by 37% (81/220), kidney disease alone by 3% (6/220), and both by 49% (109/220). High-risk genotypes were more common among those with both hypertension and kidney disease in their family (18%) versus those without either (12%). High-risk *APOL1* genotype prevalence rose with increasing antihypertensive medication use (24% among those prescribed ≥4 agents) and was higher among individuals with lower race-free estimated glomerular filtration rate (eGFR) or albuminuria. Among 173 participants with eGFR values at 3-months, high-risk genotypes were more frequent among those with eGFR decline versus improvement (23% vs. 12%).

**Conclusion:**

*APOL1* high-risk genotypes were more common among individuals with a family history of hypertension and/or kidney disease, greater medication burden, lower kidney function, and early eGFR decline. The partial overlap between genetic and familial risk underscores the complex interplay of inherited, clinical, and environmental factors. Continued study and integrative strategies are needed to refine kidney disease risk stratification, enhance early identification, and guide prevention and treatment in at-risk populations.

## Introduction

Chronic kidney disease (CKD) is a complex condition with many causes, among which genetic contributions are increasingly recognized, particularly in patients with a family history of CKD or groups with ancestry-based risk. Besides having an increased clustering of end stage kidney disease (ESKD) in families (1), Black individuals develop CKD and ESKD more often than White individuals (2). While some of the CKD risk among Black families is related to comorbid hypertension, diabetes mellitus, and environmental and social determinants of health (2), apolipoprotein L1 gene (*APOL1*) variants have been identified as significant risk factors for non-diabetic kidney disease and CKD, most of which were previously attributed to hypertensive nephropathy in this population (3, 4). Thus, a recently published report from a Kidney Disease: Improving Global Outcomes (KDIGO) Controversies Conference uses the term “*APOL1* kidney disease’’ to describe kidney pathologies associated with *APOL1* G1 and G2 renal risk variants (RRVs) (5). Identification of *APOL1* RRVs has fostered growing interest in the role of genetic testing in assessing and managing CKD risk among increased-risk populations, particularly Black individuals (6, 7).

*APOL1* encodes the apolipoprotein L1 protein, and two RRVs (G1 and G2) are almost exclusively present in individuals of sub-Saharan African descent, but virtually absent in those of European ancestry and other non-African populations. Individuals who inherit 2 *APOL1* RRVs (G1/G1, G1/G2, or G2/G2) are considered to have high-risk genotypes. While *APOL1* high-risk genotypes have been associated with faster decline in kidney function and higher ESKD risk (8, 9), only about 20% of individuals with high-risk *APOL1* genotypes develop kidney failure. While the mechanism(s) of injury associated with *APOL1* high-risk genotypes or approved targeted therapies remain to be fully elucidated (7), several clinical trials investigating potential therapies for *APOL1* kidney disease are being conducted (10).

As genetic testing for CKD becomes more accessible and *APOL1*-targeted therapies emerge, understanding the distribution of *APOL1* high-risk genotypes in Black individuals, who are at increased risk for CKD and ESKD, is critical. Notably, *APOL1* high-risk genotypes have been reported in approximately 15% of Black Americans in some samples (3, 6), but the prevalence likely varies with clinical traits and may vary with family history. We thus sought to examine the distribution of *APOL1* RRVs in relation to baseline clinical factors and family history among Black individuals receiving care at a Midwestern academic medical center. Clarifying these patterns, including the interplay with family history, may help identify opportunities for targeted education and kidney disease prevention, including opportunities for participation in interventional studies and focused work on risk communication (11). This current study investigated the *APOL1* RRV distribution in relation to family history and baseline clinical factors, and patterns of renal function change, in Black individuals who underwent *APOL1* genotyping and completed 3-month follow-up after enrollment.

## Methods

### Study design and participants

We conducted a single-center, prospective study of self-identified Black individuals seen by Nephrology and Internal Medicine services at a Midwestern academic medical center. Eligibility criteria included self-identified Black race, age ≥18 years and not on dialysis or with a kidney transplant at enrollment. Family and household members of enrolled patients were also added to eligibility on 2/23/2023. Potential participants received an explanation of *APOL1* and current understanding of the relationship of RRVs with CKD. Written informed consent was obtained prior to genetic testing, and participants received an honorarium for their time at enrollment and with survey completion. Baseline enrollment included blood sample collection for *APOL1* genetic testing, chart review of demographics and parameters related to kidney health [age, sex, body mass index (BMI), blood pressure, anti-hypertensive medications (if any), diabetes mellitus, diabetes medications (if any)], and administration of surveys including information on sociodemographic factors and family history (additional survey data such as attitudes will be reported separately).

Participant surveys, chart review of health history and laboratory data were performed at baseline, and approximately 3-months and 12-months after enrollment, in coordination with standard of care follow-up. The genetic test was the only study-specific laboratory assessment performed at enrollment. Other clinical laboratory parameters were abstracted from the medical record, as available, from participants’ routine clinical care. Enrolled participants who did not return for follow-up at the hospital had the opportunity to complete surveys by email or telephone. The study was approved by the Saint Louis University Institutional Review Board (Protocol ID 29188) and is registered under ClinicalTrials.gov (NCT05656261) (12). Enrollment began in 01/24/2019, and this report analyzes a sample who completed 3-month post-enrollment follow-up as of 03/21/2025.

### Sociodemographic characteristics and family history

Sociodemographic characteristics included age and sex, educational attainment, and self-reported annual income. The family history of hypertension and family history of kidney disease were assessed per the enrolled participant’s report on the survey.

### Genetic testing

**Figure 1:**
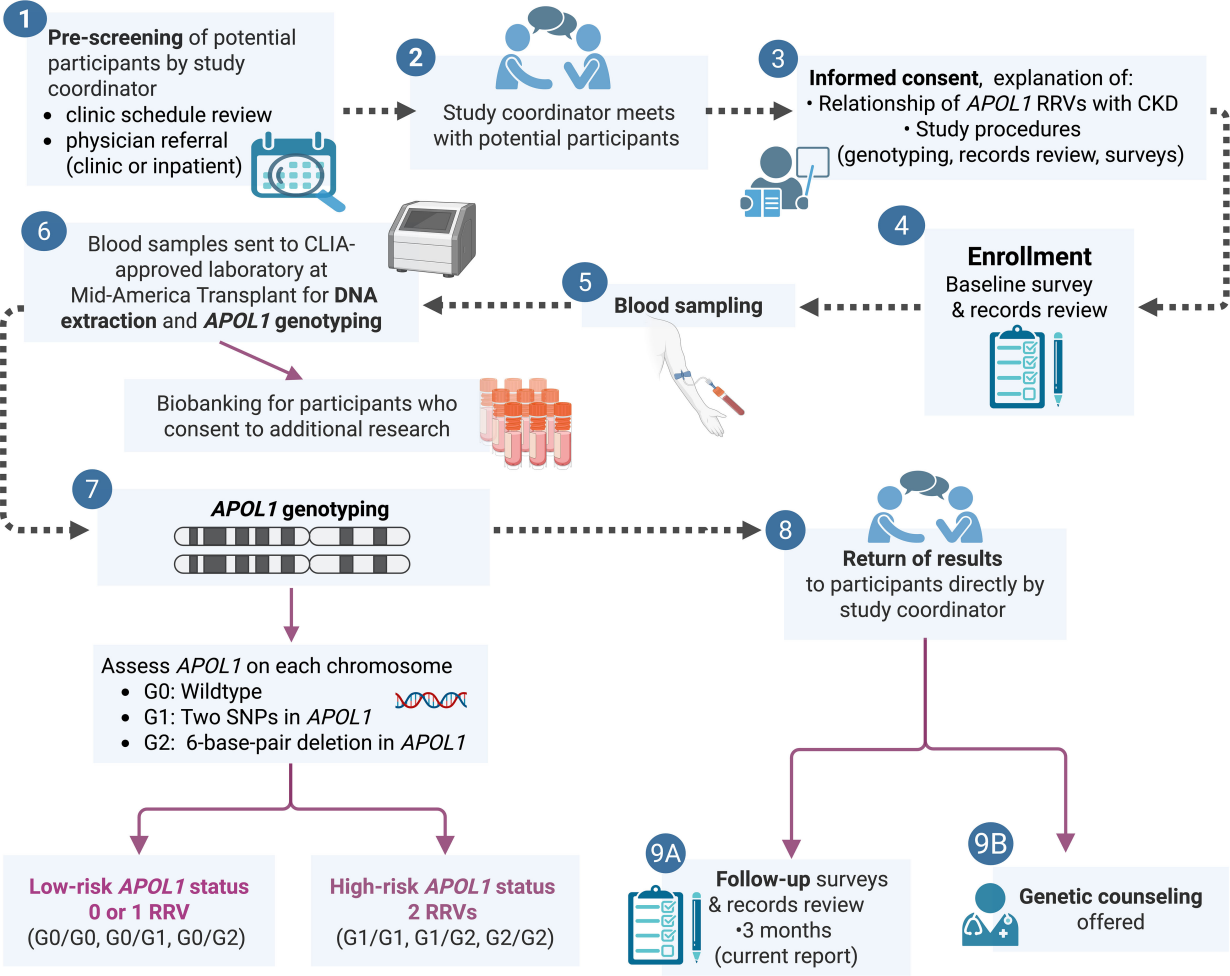
Schematic presentation of study procedures. *APOL1*, apolipoprotein L1; CKD, chronic kidney disease; CLIA, Clinical Laboratory Improvement Amendments; DNA, deoxyribonucleic acid; RRV, renal risk variant; SNP, single nucleotide polymorphism. *Surveys will be reported separately. Created in https://BioRender.com.

After the baseline survey at enrollment, blood samples were drawn and sent to the CLIA-approved lab at Mid-America Transplant for *APOL1* genotyping. Two single nucleotide polymorphisms (SNPs) in the *APOL1* G1 renal-risk allele (rs73885319; rs60910145) and an insertion/deletion for the G2-renal-risk allele (rs143830837) were genotyped using a custom assay designed at Wake Forest University School of Medicine on the Sequenom platform (San Diego, California) (13). *APOL1* genotype results were expressed as 0 *APOL1* RRV (wildtype), 1 *APOL1* RRV (G1 or G2 allele), or 2 *APOL1* RRVs (14). An *APOL1* low-risk genotype was defined as the presence of 0 or 1 RRV, meaning no variant (G0/G0) or a monoallelic variant (G0/G1 or G0/G2). *APOL1* high-risk genotypes were defined as the presence of 2 RRVs, meaning biallelic variants (G1/G1, G2/G2, or G1/G2). Patients with *APOL1* high-risk genotypes were counseled by a nephrologist on the study team and were also offered additional genetic counseling sessions with a genetic counselor at our center. Research results were returned to the participants before the second survey. Results were confidential and were not entered into the medical record, but patients could discuss with their treating physician if they chose to do so. The flow of study procedures is illustrated in **Figure 1**.

### Clinical and laboratory data

The presence of anti-hypertensive medications (if hypertensive), diabetes and antidiabetic medications (if diabetic), BMI and laboratory parameters [including fasting blood glucose, serum creatinine, estimated glomerular filtration rate (eGFR), hemoglobin, lipid profile, serum uric acid, urine albumin-to-creatinine ratio (UACR) and urine protein-to-creatinine ratio (UPCR)] were obtained from the electronic health records at the time of enrollment and at 3-month follow-up. eGFR was calculated by the 2021 race-free CKD-EPI creatinine equation (15). Urine protein-to-creatinine ratio was converted to UACR using the published formula:

*UACR (mg/mg) = -0.171 + 0.780 x UPCR (mg/mg)* (16).

### Statistical analysis

Demographic and clinical parameters were summarized according to *APOL1* genotype, presented as a percentage (%) and compared with the Chi-square test for categorical variables. Continuous variables were presented as mean ± standard deviation and were compared with the Wilcoxon Rank Sum test. *APOL1* RRV distribution percentages were examined according to baseline clinical traits. *APOL1* RRVs and family history of hypertension and/or family history of kidney disease distributions were examined across baseline eGFR levels. Finally, *APOL1* RRVs and family history of hypertension and/or family history of kidney disease distributions were also examined according to eGFR level change patterns from baseline to 3-month follow-up. For the follow-up analysis, eGFR levels were defined as: ≥60, 45 to 59, 30 to 44 or <30 ml/min/1.73 m^2^, and change patterns were categorized as ‘eGFR decreased’, ‘eGFR stayed same’ or ‘eGFR increased’.

Differences were considered statistically significant if the two-tailed p-value was <0.05. However, in general, statistical significance was not anticipated due to the sample size.

## Results

### Baseline sociodemographic characteristics and laboratory data

Between 2019 to March 2025, 220 enrolled participants met eligibility criteria for the current analysis. Median age of the patients was 59 years, and 54% were women (119/220). Median BMI was 32.7 kg/m^2^ and 22% had diabetes mellitus. In the cohort, 83% (183/220) had an education of high school equivalent or higher, and 62% (136/220) reported an annual family income ≤$45,000 (**Table 1**).

**Table 1:** Baseline sociodemographic characteristics and clinical data of the study cohort grouped by *APOL1* low- and high-risk genotypes.

Baseline factor	Overall (n=220)	Low-risk genotype (n=183)	High-risk genotype (n=37)
Demographic traits, median (IQR)			
Age, years	59 (17)	59 (17)	57 (18)
Body mass index, kg/m^2^	32.7 (9.9)	32.4 (9.6)	34.2 (11.0)
Sex, n (%)			
Female	119 (54)	102 (56)	17 (46)
Male	101 (46)	81 (44)	20 (54)
Educational attainment, n (%)			
Less than eighth grade	5 (2)	4 (2)	1 (3)
More than eighth grade but less than high school	29 (13)	22 (12)	7 (19)
High school graduate or GED	66 (30)	59 (32)	7 (19)
Some college or technical school	63 (29)	55 (30)	8 (21)
College graduate or greater	54 (25)	40 (22)	14 (38)
Not reported, n (%)	3 (1)	3 (2)	0 (0)
Annual family income, n (%)			
<$5,000	43 (19)	37 (20)	6 (16)
>$5,000 to $15,000	30 (14)	25 (14)	5 (14)
>$15,000 to $30,000	33 (15)	27 (15)	6 (16)
>$30,000 to $45,000	30 (14)	24 (13)	6 (16)
>$45,000 to $60,000	23 (10)	16 (9)	7 (19)
>$60,000	28 (13)	26 (14)	2 (5)
Not reported, n (%)	33 (15)	28 (15)	5 (14)
Family history n (%)			
Hypertension alone	81 (37)	68 (37)	13 (35)
Kidney disease alone	6 (3)	5 (3)	1 (3)
Hypertension and kidney disease	109 (49)	89 (49)	20 (54)
Neither	24 (11)	21 (11)	3 (8)
Blood pressure, mm Hg, median (IQR)			
Systolic	138 (25)	139 (24)	137 (24)
Diastolic	82 (18)	82 (18)	81 (12)
Blood pressure medications, n (%)			
ACE inhibitor	53 (24)	46 (25)	7 (19)
Angiotensin receptor blocker	78 (37)	65 (36)	13 (35)
Beta blocker	80 (36)	64 (35)	16 (43)
Calcium channel blocker	116 (53)	93 (51)	23 (62)
Thiazide diuretic	53 (24)	44 (24)	9 (24)
Loop diuretic	31 (14)	22 (12)	9 (24) *
Vasodilator	30 (14)	22 (12)	8 (22)
Other	43 (20)	33 (19)	8 (22)
Number of blood pressure medications, median (IQR)	2 (2)	2 (2)	3 (3)
Diabetes status, n (%)			
Diabetes	49 (22)	41 (22)	8 (22)
Diabetes mellitus medications, n (%)			
Metformin	14 (6)	11 (6)	3 (8)
SGLT2 inhibitor	23 (11)	20 (11)	3 (8)
Sulfonylurea	4 (2)	3 (2)	1 (3)
GLP-1 receptor agonist	14 (6)	13 (7)	1 (3)
DPP-4 inhibitor	3 (1)	3 (2)	0 (0)
Thiazolidinedione	1 (0.5)	1 (0.5)	0 (0)
Insulin	22 (10)	19 (10)	3 (8)
Other	2 (1)	1 (0.5)	1 (2)

Percentages are presented as column percents (%).

* <0.05.

Wilcoxon Rank Sum tests for continuous variables and Chi-Square tests for proportions.

RRV, Renal risk variants; IQR, interquartile range; ACE, angiotensin-converting enzyme; GED, General educational development.

In the whole cohort, 17% (37/220) had *APOL1* high-risk genotypes and 83% (183/220) had *APOL1* low-risk genotypes. Sociodemographic and clinical characteristics were similar in those with *APOL1* low-risk and high-risk genotypes (**Table 1**). The median number of antihypertensive medications used was 2 in the *APOL1* low-risk group and 3 in the *APOL1* high-risk group. The prevalence of loop diuretic use was significantly higher in *APOL1* high-risk group when compared to *APOL1* low-risk group (P <0.05).

Baseline laboratory data of the cohort, overall and by *APOL1* genotype, are summarized in **Table 2**. Serum creatinine values were not available among 3 participants. At baseline, patients with *APOL1* high-risk genotypes had slighlty higher hemoglobin values, and similar fasting blood glucose and lipid profiles, compared to patients with *APOL1* low-risk genotypes. There was a trend for lower race-free eGFR, higher serum uric acid, and higher UACR in the *APOL1* high-risk group compared to the *APOL1* low-risk group, although these trends did not reach statistical significance. Participants with high-risk genotypes tended to have lower kidney function at baseline compared with the low-risk genotype group, with a median eGFR of 43.0 mL/min/1.73 m^2^ (IQR 29.6) versus 51.2 mL/min/1.73 m^2^ (IQR 35.7). Reduced kidney function (eGFR <45 mL/min/1.73 m^2^, encompassing the 30 to 44 and <30 mL/min/1.73 m^2^ categories), was more common among participants with high-risk genotypes (54.0%) compared to those with low-risk genotypes (42.0%). Advanced kidney impairment (eGFR <30 mL/min/1.73 m^2^) was also more frequent at baseline in the high-risk genotype group (24.3% vs 16.9%). In contrast, preserved kidney function (eGFR ≥60 mL/min/1.73 m^2^) was less common among individuals with high-risk genotypes (27.0% vs 38.8%).

**Table 2:** Baseline laboratory data of the study cohort grouped by *APOL1* RRV.

Baseline laboratory tests	Overall	Low-risk genotype ^a^	High-risk genotype
Serum creatinine, mg/dL, median (IQR), n=217	1.4 (0.8)	1.4 (0.9)	1.6 (1.0)
eGFR, ml/min/1.73 m^2^, median (IQR), n=217	50.0 (35.4)	51.2 (35.7)	43.0 (29.6)
eGFR levels, ml/min/1.73 m^2^, n (%)			
≥60, n= 81	81 (36.8)	71 (38.8)	10 (27.0)
45 to 59, n= 39	39 (17.7)	32 (17.5)	7 (18.9)
30 to 44, n= 57	57 (25.9)	46 (25.1)	11 (29.7)
<30, n=40	40 (18.2)	31 (16.9)	9 (24.3)
Not reported, n=3	3 (1.4)	3 (1.6)	0 (0)
Albuminuria, mg/g, median (IQR)			
Urinary albumin-to-creatinine ratio, n=135	33 (335)	29 (335)	86.0 (366)
Serum lipids, mg/dL, median (IQR)			
Total cholesterol, n=145	166 (49)	168 (53)	157 (59)
High density lipoprotein cholesterol, n=145	47 (22)	47 (22)	47 (20)
Low-density lipoprotein cholesterol, n=143	95 (46)	97 (43)	82 (42)
Triglycerides, n=144	103 (75)	106 (73)	100 (90)
Other metabolic tests, median (IQR)			
Fasting blood glucose mg/dL, n=107	89 (28)	101 (31)	99 (28)
Serum uric acid mg/dL, n=118	6.7 (2.6)	6.7 (2.2)	7.4 (3.3)
Hemoglobin g/dL, n=107	12.4 (2.3)	12.4 (2.1)	13.0 (3.4) *

Wilcoxon Rank Sum tests for continuous variables.

*<0.0001.

RRV, Renal risk variants; IQR, interquartile range; eGFR, estimated glomerular filtration rate.

^a^ Three patients in the low-risk group did not have serum creatinine data.

### *APOL1* RRV distributions according to baseline sociodemographic and clinical factors

**Figure 2:**
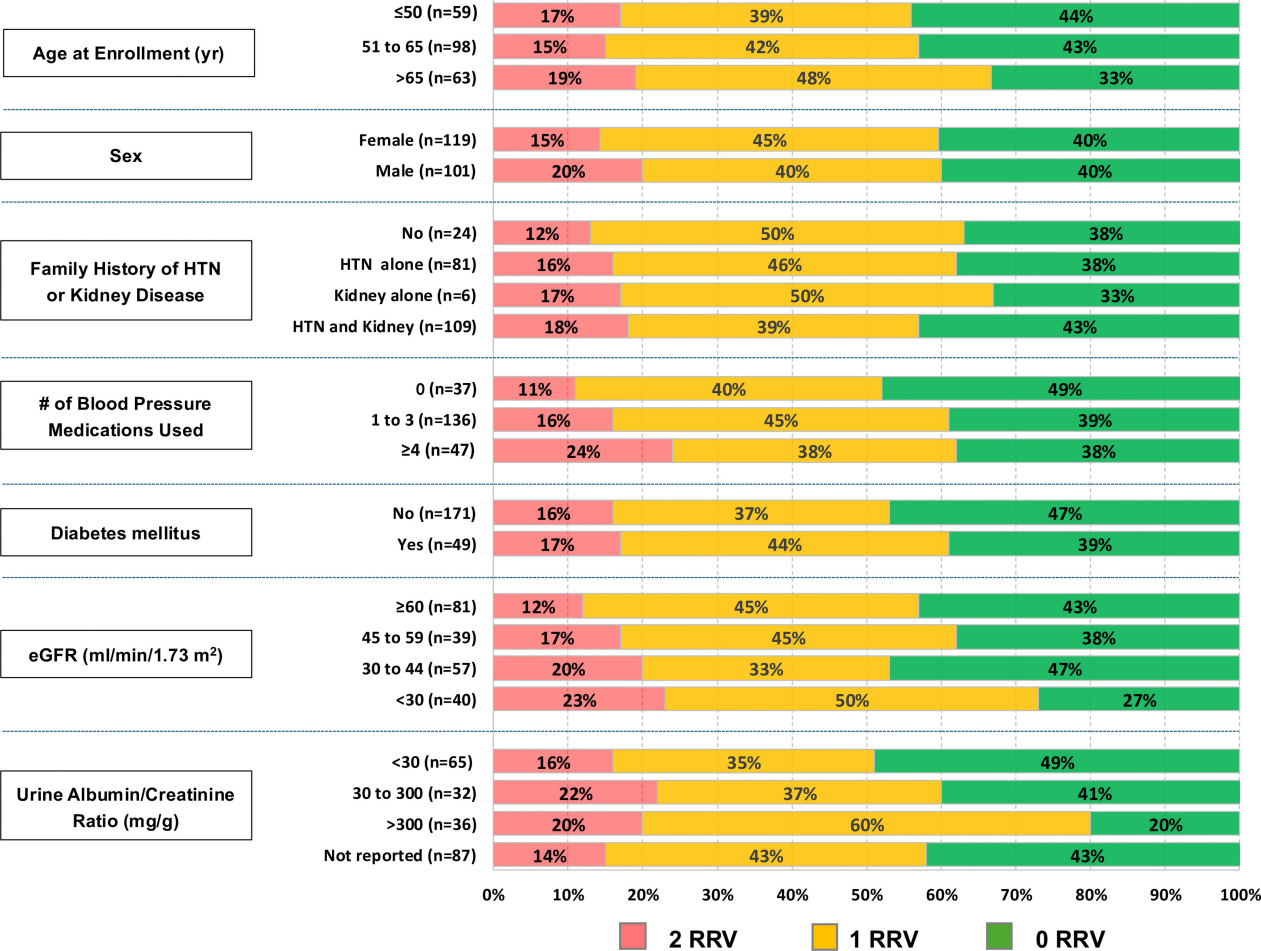
Distribution of *APOL1* RRV by baseline characteristics (n =220). *APOL1*, apolipoprotein L1; RRV, renal risk variant; HTN, hypertension; eGFR, estimated glomerular filtration rate (ml/min/1.73 m^2^).

The majority of patients were aged between 51 to 65 years (45%; 98/220); 27% (59/220) were ≤50 years of age while 29% (63/220) were older than age 65. Across the age ranges, the percentage of patients with 2 *APOL1* RRVs ranged between 15 to 19% and the percentage with high-risk genotypes were highest amongst participants who were >65 years of age (19%; 12/63) (**Figure 2**). The percentage of patients with 1 *APOL1* RRV showed a graded increase from 39% (23/59) in patients ≤50 years of age to 48% (30/63) in patients who were >65 years of age. Accordingly, the percentage of patients with 0 *APOL1* RRVs showed a graded decrease as the patient's age range increased. There was a nonsignificant female predominance in the *APOL1* low-risk group (56% vs 44%), and male predominance in the *APOL1* high-risk group (54% vs 46%) (**Table 1**). The proportion with high-risk genotypes was higher among male patients (20%; 20/101) than among female patients (15%; 17/119), although the difference was not statistically significant (**Figure 2**).

At baseline, 37% (81/220) of patients reported a family history of hypertension (without kidney disease), 3% (6/220) reported a family history of kidney disease (without hypertension), and 49% (109/220) reported a family history of both hypertension and kidney disease. In the whole cohort, 11% (24/220) of the patients reported no family history of hypertension or kidney disease (**Table 1**). The prevalence of *APOL1* high-risk genotypes showed a mildly graded increase amongst patients who reported a family history of hypertension (16%; 13/81), kidney disease (17%; 1/6) or both (18%; 20/109). In patients without a family history of either condition, only 12% (3/24) had an *APOL1* high-risk genotype. *APOL1* low-risk genotypes were most common (88%; 21/24) in patients who did not report a family history of hypertension and/or kidney disease (**Figure 2**).

The prevalence of high-risk *APOL1* genotypes showed a graded increase with higher antihypertensive medication requirements, from 11% (4/37) in patients who were not taking any antihypertensive medications to 24% (11/47) in those taking ≥4 agents. The prevalence of 0 *APOL1* RRVs was highest (49%; 18/37) amongst patients who were not taking any antihypertensive medications (**Figure 2**). The prevalence of *APOL1* high-risk genotypes was similar in those with and without diabetes mellitus [17% (8/49) vs. 16% (27/171)]. In the full cohort, 11% (23/220) of the patients were receiving a sodium glucose co-transport-2 inhibitor (SGLT2i): three of these patients had *APOL1* high-risk genotypes, and all three had diabetes.

The prevalence of *APOL1* high-risk genotypes showed a graded increase as the baseline eGFR decreased, from 12% (10/81) in those with eGFR ≥60 ml/min/1.73 m^2^ to 23% (9/40) in those with eGFR <30 ml/min/1.73 m^2^. Conversely, the prevalence of *APOL1* low-risk genotypes showed a graded decrease as the baseline eGFR decreased, from 88% (71/81) in those with eGFR ≥60 ml/min/1.73 m^2^ to 77% (31/40) in those with eGFR <30 ml/min/1.73 m^2^ (**Figure 2**).

*APOL1* high-risk genotypes were more common in patients who had albuminuria [22% (7/32) in those with UACR 30 to 300 mg/g and 20% (7/36) in those with UACR >300 mg/g] than in those who had normo-albuminuria [16% (10/65) in those with UACR <30 mg/g]. In contrast, the *APOL1* low-risk genotype prevalence was highest in patients with UACR <30 mg/g (84%; 55/65). Albuminuria measurement was performed in routine care and was not available in 87/220 (40%) of the cohort.

### *APOL1* RRV and family history across different levels of baseline eGFR

**Figure 3:**
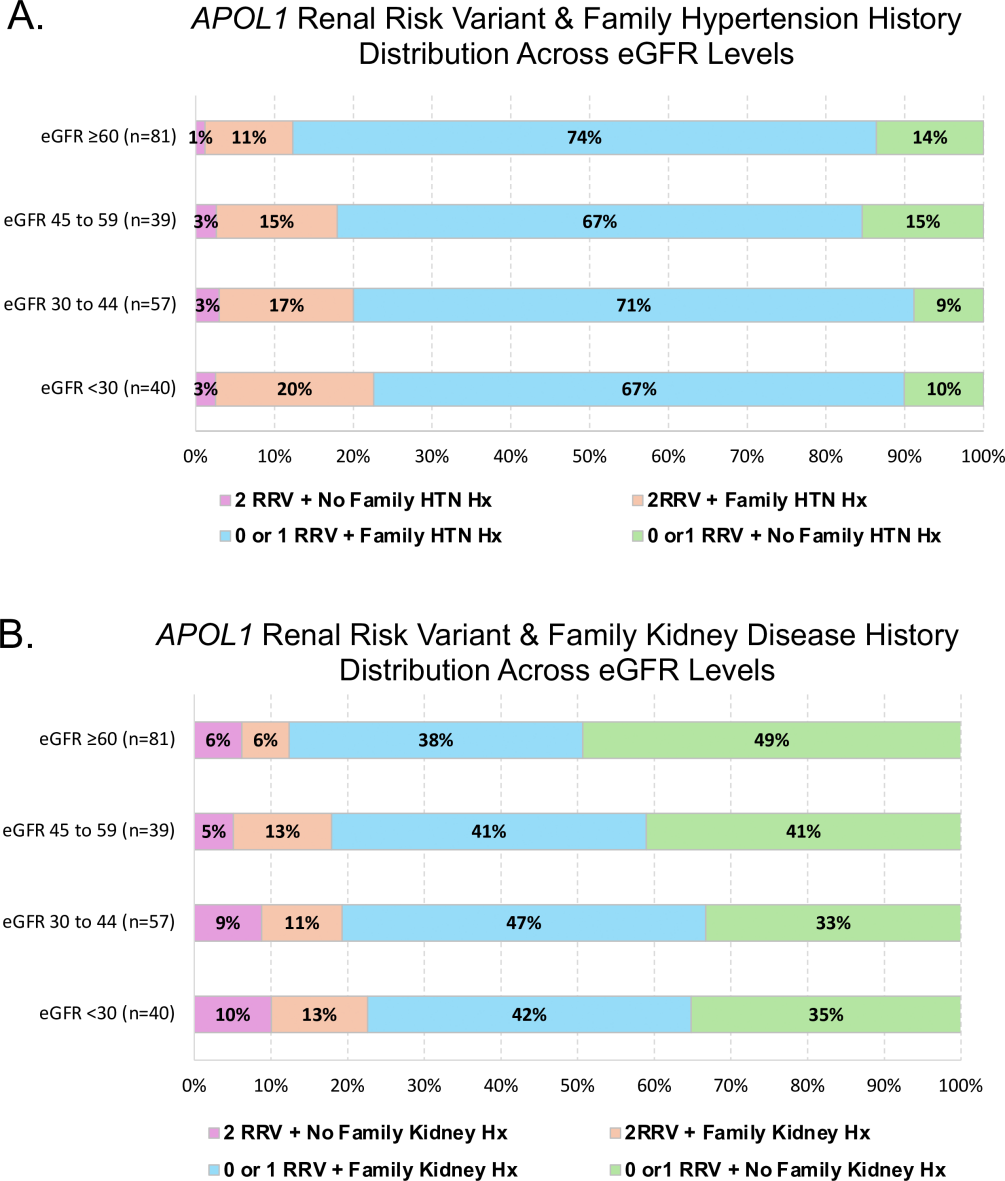
Distribution of *APOL1* RRV and family history of hypertension **(A)** and kidney disease **(B)** across eGFR levels. *APOL1*, apolipoprotein L1; RRV, renal risk variant; eGFR, estimated glomerular filtration rate (ml/min/1.73 m^2^); HTN, hypertension; Hx, history.

A family history of hypertension was common in the study cohort and was present in 85% (69/81) of the patients with eGFR ≥60 and in 87% (35/40) patients with eGFR <30 ml/min/1.72 m^2^. Consideration of *APOL1* genotype and family history of hypertension across different baseline eGFR levels showed that most patients with *APOL1* high-risk genotypes also had a family history of hypertension; only 1-3% of patients overall had a high-risk genotype without a family history of hypertension (**Figure 3A**). Patients with a family history of hypertension and *APOL1* high-risk genotypes showed a graded increase with progressively lower baseline eGFR, from 11% (9/81) in those with eGFR ≥60 ml/min/1.73 m^2^ to 20% (8/40) in those with eGFR <30 ml/min/1.73 m^2^ (**Figure 3A**).

A family history of kidney disease was present in 44% (36/81) with eGFR ≥60 ml/min/1.73 m^2^ and 55% (22/40) with eGFR <30 ml/min/1.73 m^2^ (**Figure 3B**). More than half of patients with *APOL1* high-risk genotypes reported a family history of kidney disease, with or without hypertension (**Table 1**). Among those with eGFR ≥60 ml/min/1.73 m^2^, 6% had high-risk genotypes with a family history of kidney disease and 6% had high-risk genotypes without a family history of kidney disease. At lower eGFR levels, 11% to 13% had high-risk genotypes and a family history of kidney disease across categories, while lower proportions had high-risk genotypes without a family history of kidney disease (**Figure 3B**).

### *APOL1* RRV distributions according to eGFR change from baseline to 3 months

**Figure 4:**
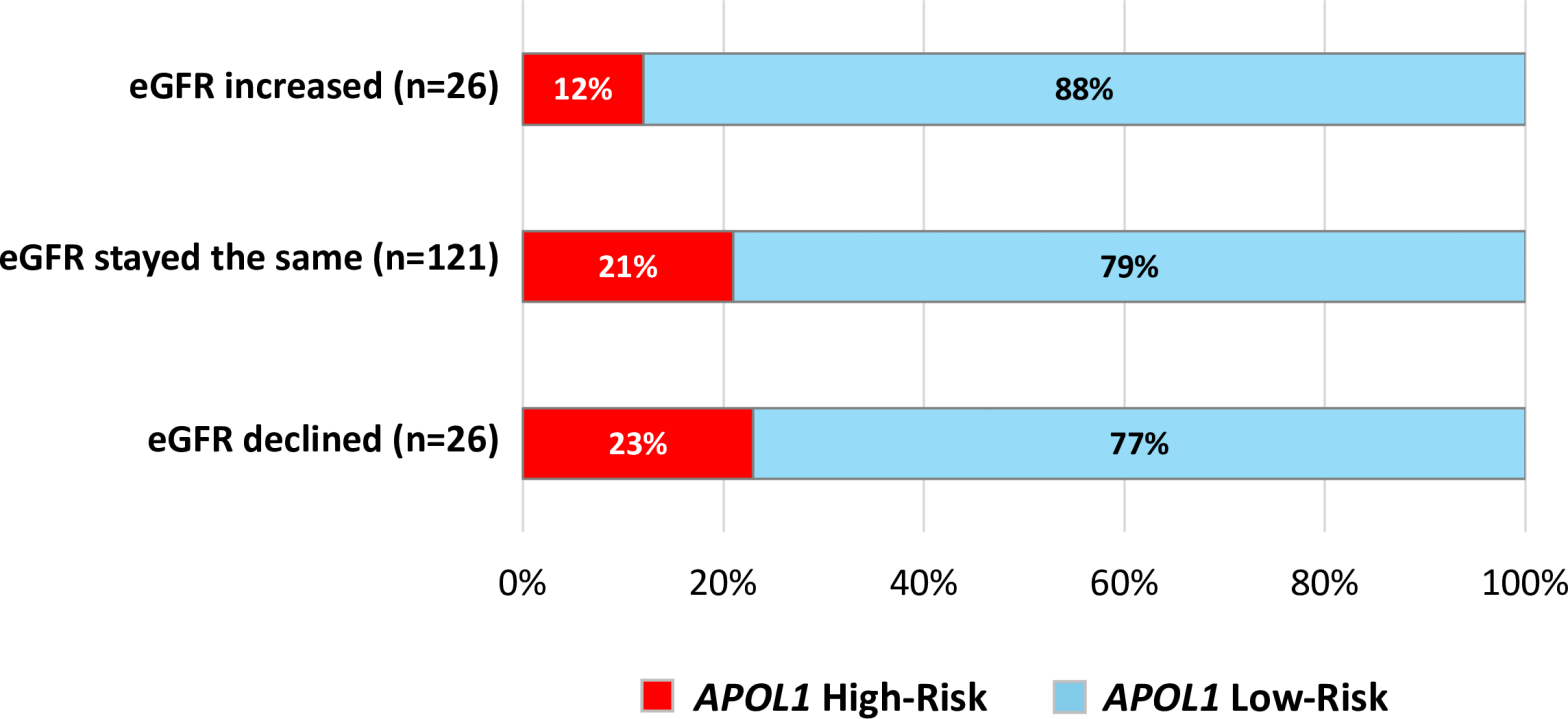
Distribution of *APOL1* RRV across eGFR changes from baseline to 3 months. *APOL1*, apolipoprotein L1; eGFR, estimated glomerular filtration rate (ml/min/1.73 m^2^).

In the cohort, 173 patients had serum creatinine and eGFR values measured at both baseline and 3-months. eGFR was stable within the same level in the majority of patients at 3 months (70%; 121/173), while 15% (26/173) experienced an increase to a higher eGFR level, and 15% (26/173) experienced a decline to a lower eGFR level (**Figure 4**). *APOL1* high-risk genotypes were found in 23% (6/26) of those who experienced a decline in eGFR level, but only in 12% (3/26) of those whose eGFR level increased.

## Discussion

The distribution of *APOL1* genotype and self-reported family history of hypertension and kidney disease were examined in Black individuals at a Midwestern, academic medical center. Several key observations emerged. First, 17% of participants had *APOL1* high-risk genotypes. Nearly half reported a family history of both hypertension and kidney disease, while 37% reported a family history of hypertension alone and 3% had a family history of kidney disease alone. Second, *APOL1* high-risk genotypes were more prevalent among individuals with a family history of hypertension and/or kidney disease, as well as those with a greater antihypertensive medication burden, lower kidney function, albuminuria, and evidence of early decline in eGFR. Third, genetic and familial risk factors only partially overlapped. For example, among participants with eGFR <30 ml/min/1.73m^2^, 23% had high-risk genotypes; of these 56% also had a family history of kidney disease (13% of those in this eGFR level), while 44% did not (10% of those in this eGFR level). These findings underscore the need for further research to disentangle the genetic and environmental components of familial risk and to guide targeted interventions based on individualized risk profiles.

Patients with *APOL1* high-risk genotypes in the cohort had similar sociodemographic and clinical characteristics to those with *APOL1* low-risk genotypes. There was a nonsignificant male predominance in the *APOL1* high-risk group, while females predominated in the *APOL1* low-risk group. We observed a trend for lower eGFR and higher albuminuria in patients with *APOL1* high-risk genotypes when compared to those with *APOL1* low-risk genotypes. This observation aligns with existing evidence that *APOL1* high-risk genotypes pose a higher risk of developing CKD and various forms of kidney disease (17), aka *APOL1* kidney disease (5), including focal segmental glomerulosclerosis, HIV-associated nephropathy, pre-eclampsia and faster progression to ESKD (17) in Black individuals.

*APOL1* high-risk genotypes are associated with increased risk of CKD development and progression to ESKD (18) and increase the risk of developing non–diabetic CKD by up to 7 to 10 fold in some studies (17). In our cohort, we observed that patients with *APOL1* high-risk genotypes, who also tended to have a lower eGFR, needed a greater antihypertensive pill burden in their clinical management when compared to those with *APOL1* low-risk genotypes. This may reflect the impacts of lower renal function associated with *APOL1* kidney disease. As emphasized in a recent KDIGO Controversies Conference on Genetics in CKD, it is important not to misclassify *APOL1* kidney disease as hypertensive nephropathy (19). Notably, in a secondary analysis of the African American Study of Kidney Disease and Hypertension (AASK) and the Chronic Renal Insufficiency Cohort (CRIC) study participants, Black individuals with *APOL1* high-risk genotypes had significantly higher risk of CKD progression than those with *APOL1* low-risk genotypes, and the effect of *APOL1* high-risk genotypes on CKD progression was independent of blood pressure and anti-hypertensive medication class (20). Traditionally, *APOL1* kidney disease was solely linked to the presence of two *APOL1* RRVs (biallelic variants). However, Gbadegesin at al recently reported that even carrying a single *APOL1* RRV (monoallelic variant) significantly increases the risk of CKD and focal segmental glomerulosclerosis in West African populations (21). Thus, we believe that future studies investigating whether *APOL1* monoallelic variants predispose to kidney damage in populations outside Africa are warranted.

*APOL1* high-risk genotypes are less clearly associated with diabetic kidney disease (DKD). Rather, type 2 diabetes mellitus and *APOL1* kidney disease are common entities that may co-exist in some individuals. *APOL1* high-risk genotypes were associated with an increased rate of progression of DKD in some studies (10). In our study cohort, the prevalence of diabetes mellitus was not significantly different across the *APOL1* genotypes. Whether novel therapeutic advances in *APOL1* kidney disease may slow the progression of DKD in Black individuals is unknown at this time, mostly because early trials focusing on the potential treatments for *APOL1* kidney disease excluded patients with diabetes mellitus (22). More recent trials testing the safety and efficacy of novel potential therapies for *APOL1* kidney disease are including patients with diabetes mellitus (NCT06830629, NCT06824987), allowing for equity in access in trial participation for diabetic patients.

Currently, there is no curative treatment for *APOL1* kidney disease. However, novel therapies targeting *APOL1* kidney diease are under development. Inaxaplin (VX-147), a selective, oral, small-molecule inhibitor of *APOL1* channel function has been shown to reduce proteinuria in patients with *APOL1* high-risk genotypes and biopsy-proven focal segmental glomerulosclerosis (22). The effects of MZE829, an *APOL1* pore inhibitor, on albuminuria in patients with *APOL1* kidney disease is being tested in a Phase 2 trial (NCT06830629) and an *APOL1* anti-sense oligonucleotide is also under study (NCT06824987). The antiproteinuric efficacy and safety of JAK1/2 inhibition with baricitinib, an oral JAK1/2-specific inhibitor that blocks APOL1 synthesis, is currently under investigation in patients with *APOL1*-associated focal segmental glomerulosclerosis and *APOL1*-associated hypertension-attributed CKD (23). Recently, SGLT2is have emerged as highly effective therapies in non-diabetic proteinuric kidney diseases. In *APOL1* kidney disease, many pathologic processes including, but not limited to, mitochondrial dysfunction, inflammation and disordered autophagy may be targets for SGLT2is (24). Thus, we believe that there is a rationale to investigate the effects of SGLT2is on *APOL1* kidney disease in future studies (24). In our cohort, 11% patients were taking a SGLT2i: three of these patients had *APOL1* high-risk genotypes and all three of these patients were diabetic.

There are ongoing public efforts to acknowledge *APOL1* kidney disease in kidney care. Recently, the Kidney Health Initiative, a public-private partnership of the American Society of Nephrology and the US Food and Drug Administration, released a roadmap to increase awareness of *APOL1* kidney disease, increase access to and awareness of genetic testing and associated counseling, and empower patients with *APOL1* kidney disease to make informed decisions about participating in clinical studies (25). *All of Us* is part of a new era in which researchers, health care providers, technology experts, community partners, and the public work together to develop individualized health care. This program aims for in-depth genetic screening for kidney disease and seeks potential new ways to protect against or treat kidney disease (26). These initiatives seek to identify high-risk participants for recruitment to research in *APOL1* kidney disease.

In our study cohort, the prevalence of *APOL1* high-risk genotypes trended higher amongst patients who reported a family history of hypertension and/or kidney disease. We observed a similar trend in the distribution of *APOL1* high-risk genotypes across eGFR levels: *APOL1* high-risk genotypes were more common at lower baseline eGFR levels. Thus, there was a partial overlap between *APOL1* high-risk genotypes, family history and individual CKD. However, most of the patients with a family history of hypertension and/or kidney disease did not carry *APOL1* high-risk genotypes. Our findings underscore the need for untangling the complex interplay between genetics and environmental factors in *APOL1* kidney disease in Black individuals (27).

Our study has limitations. The sample size of our cohort to date is small, which limits statistical power. However, it is critical to note that data were assembled in a unique, prospective protocol in which individual patients were educated about *APOL1* kidney disease and consented to participation. While such an approach is more time-consuming and labor-intensive than genotyping among biobanks assembled for other purposes, the study also served the community in providing education about *APOL1* kidney disease to patients and families. The 3-month follow-up duration provides pilot data, but is too short to draw robust conclusions about assocations of *APOL1* RRVs and outcomes. The study was conducted at an urban Midwestern U.S. medical center, which may not be generalizable to rural areas or whole U.S. Black population. Notably, our cohort included adults age 65 years and older, capturing an understudied age group, although younger patients were less common.

Despite the accumulating data about the association between *APOL1* high-risk genotypes and the development and progression of CKD, the results of *APOL1* genetic testing are not immediately actionable at the present time, given the lack of FDA-approved therapeutic options that target *APOL1* kidney disease. There may be heterogeneity in the attitudes, knowledge and enthusiasm across nephrologists caring for Black individuals to test for *APOL1* RRVs, impacting the implementation of widespread *APOL1* genetic testing. Outside of a study protocol, *APOL1* testing might pose a financial burden for some of patients at this time, although notably a billing code for *APOL1* kidney disease was recently introduced (28).

In conclusion, in this prospectively enrolled genotyping study, *APOL1* high-risk genotypes were more common among individuals with a family history of hypertension and/or kidney disease, greater medication burden, lower kidney function, and early eGFR decline. The partial overlap between genetic and familial risk underscores the complex interplay of inherited, clinical, and environmental factors. Importantly, this work extends beyond focus group discussions of patient interest in genotyping to illustrate actual participation in a genetic testing study. Ongoing work with this accruing cohort will include longitudinal follow-up and examination of participant attitudes toward genotyping and factors influencing kidney risk perception. Continued study in cohorts such as this is needed to clarify the genetic and environmental components of familial kidney risk, enhance early identification, and empower individuals to act upon their personalized risk profiles. As novel therapies targeting *APOL1* kidney disease are developed and clinical trials expand, increased use of genotyping may enable earlier identification of individuals at higher genetic risk who could benefit from enhanced monitoring, risk stratification, and participation in emerging therapeutic studies. In this context, education and genotyping may serve an important role in preparing patients and clinical systems for future precision-medicine approaches to kidney disease.

## Data Availability

Data are available in summary form due to limitations of the IRB. Further inquiries can be directed to the corresponding author/s.

## St. Louis APOL1 study collaborators

Orhun Aydin, PhD, Ezequiel Bellorin Font, MD, Ava DeLonais-Parker, B.S., Nourhan Houjeij, MD, Kevin Lee, MPA, Kunal Malhotra, MD, Rushda Mansuri, MD, MPH, Marilyn Maxwell, MD, Mowafaq Said, MD, Farzana Hoque, MD, Amy K. Mosman, PA, Anne Buganski, RN, Mary Lesko, RN, Kenan Li, PhD, Enbal Shacham, PhD, Than-Mai Vo, MD.
